# New Horizons in Plant–Microbe Interactions

**DOI:** 10.3390/plants13212968

**Published:** 2024-10-24

**Authors:** Martin Cerny, Veronika Hýsková

**Affiliations:** 1 Department of Molecular Biology and Radiobiology, Faculty of AgriSciences, Mendel University in Brno, 61300 Brno, Czech Republic; 2 Department of Biochemistry, Faculty of Science, Charles University, Hlavova 2030, 12843 Prague, Czech Republic

Theophrastus, an ancient Greek philosopher known as the “Father of Botany,” was among the first to document plant diseases, establishing an understanding of plant biotic interactions some 2300 years ago. However, despite the millennia that have passed since these initial observations, our grasp of the mechanisms that govern plant–pathogen interactions remains fragmentary. This knowledge gap continues to impede our ability to develop robust strategies for mitigating the detrimental effects of pests and diseases on global agriculture. Current estimates from the Food and Agriculture Organization (FAO) indicate that plant pests and diseases account for up to 40% of annual global crop loss [1], underscoring the urgent need for deeper insights and innovative solutions in this critical area of plant research. Our knowledge of plant–microbe interactions is constantly expanding, particularly thanks to advances in molecular biology and “omics” technologies, including transcriptomic, proteomic, and metabolomic analyses, all of which can be found in our Special Issue [2,3,4,5,6,7]. Thanks to advanced techniques and discoveries, the study of plant biotic interactions now extends beyond pathogenic microorganisms, pests, and herbivores that induce biotic stress, encompassing a broader range of beneficial relationships as well. This is also reflected in the composition of our Special Issue (Figure 1).

Interactions between plants and various pathogens remain among the most pressing challenges in agriculture. This Special Issue addresses one of the most devastating biotic stressors worldwide: fusarium head blight disease (FHB). FHB significantly reduces both the yield and quality of small-cereal crops like wheat and barley, while also producing mycotoxins that pose risks to global food safety [2,8]. Fiona Doohan’s team from University College Dublin discovered that the wheat NAC transcription factor TaNACL-D1 plays a crucial role in enhancing resistance to FHB. The related publication by Monika Vranić et al. [2] demonstrated that NAC overexpression led to transcriptional reprogramming and the upregulation of genes involved in hormone biosynthesis, detoxification, immune responses, secondary metabolism, and signaling, providing the putative mechanisms underlying enhanced resistance. Furthermore, their study revealed that TaNACL-D1 differentially regulates the response to jasmonic acid and abscisic acid, two hormones involved in plant stress responses. Phytohormones were also the focal point of the research article by Stephen Strelkov’s team at the University of Alberta. Jayasinghege et al. [3] profiled selected plant hormones during clubroot development in resistant and susceptible *Brassica napus* plants. Their results confirmed the previously described role of salicylic acid, jasmonic acid, and ethylene in *Plasmodiophora brassicae* infection, showing that abscisic acid levels strongly increased in susceptible plants, probably as a response to cope with clubroot-induced stress and interference with water uptake. These results further contributed to our understanding of hormonal balance in *P. brassicae*’s response. *P. brassicae* can persist in soil for over twenty years as resting spores [3,9], and pinpointing promising targets for promoting resistance against this pathogen is critical.

Bacterial zoonoses, such as salmonellosis, can be detected in plants and may threaten human health. A joint team from the Julius Kühn Institute and INRAE Val de Loire, led by Adam Schikora, studied the interaction between plant hosts and the human pathogen *Salmonella enterica*. As summarized in Han et al. [4], transcriptomics analyses compared interactions between three different *S. enterica* strains (a model strain isolated from poultry, a laboratory strain with attenuated virulence, and a strain without a virulence plasmid) and *Solanum lycopersicum*. The authors also compared these results with the available data on *Arabidopsis thaliana* and *Lactuca sativa*, showing only a limited overlap in identified pathways. These results provided a novel insight into *S. enterica* responses in plants, as well as a rich omics resource for future data mining.

New methods often open novel perspectives on classical experiments. Kopecká and Černý from Mendel University in Brno explored the composition of the xylem sap proteome and used this technique to analyze the response to the bacterial flagellin-derived elicitor flg22 in *S. tuberosum* and *H. vulgare*. A comparison with root and shoot proteome extracts highlighted the benefits of analyzing xylem sap, as many of the differentially abundant proteins identified were not significantly changed in the whole tissue proteome, including proteins of the HSP70 family [5].

New horizons are also revealing the beneficial interactions between soil microorganisms and plants. Such beneficial microorganisms, which serve as plant biostimulants, although applied in small quantities, release growth regulators. At the same time, they contribute to influencing the processes of soil nutrient solubilization and mineralization, improving their accessibility. It seems that, in the future, understanding these interactions will be crucial for environmentally friendly and sustainable crop production. The research team from the University of Copenhagen, led by Chandana Pandey, studied the potential beneficial effect of seed biopriming with biofilm-forming plant growth-promoting rhizobacteria (PGPR) on seed germination under suboptimal conditions. The interactions between four strains of PGPR from the *Pseudomonas* and *Bacillus* genera, including mutant *Pseudomonas putida* with its compromised biofilm-forming ability, and nine various accessions of *Arabidopsis thaliana* were studied. The results summarized in Pandey et al. [6] showed that biofilm-forming PGPR strains hold potential for future agricultural use due to their ability to promote plant germination and resilience against environmental stresses.

HC-Pro, a potyvirus cysteine-type protease, plays a crucial role in various stages of the viral life cycle beyond polyprotein cleavage. A joint team from Charles University and the Czech Academy of Sciences, led by Helena Ryšlavá, reviewed the state of the art in research of this interesting protein. HC-Pro aids in aphid transmission, suppresses plant defenses, and facilitates virus spread. While not typically associated with cleaving host proteins, HC-Pro has been shown to interact with several of them, potentially modulating the cellular environment to benefit the virus. Interestingly, plants have evolved mechanisms to counter HC-Pro’s effects through interactions with plant proteins. For more details and insights into these interactions, explore the review by Hýsková et al. [7].

This Special Issue covered a wide range of biotic interactions and provided new insights and ideas on how we can protect plants against biotic stress in the future. We extend our sincere gratitude to all the authors for their valuable contributions to this collection. We hope that the presented articles will serve as a rich source of knowledge, inspiration, and fresh perspectives on plant–microbe interactions for both authors and readers alike.

## Figures and Tables

**Figure 1:**
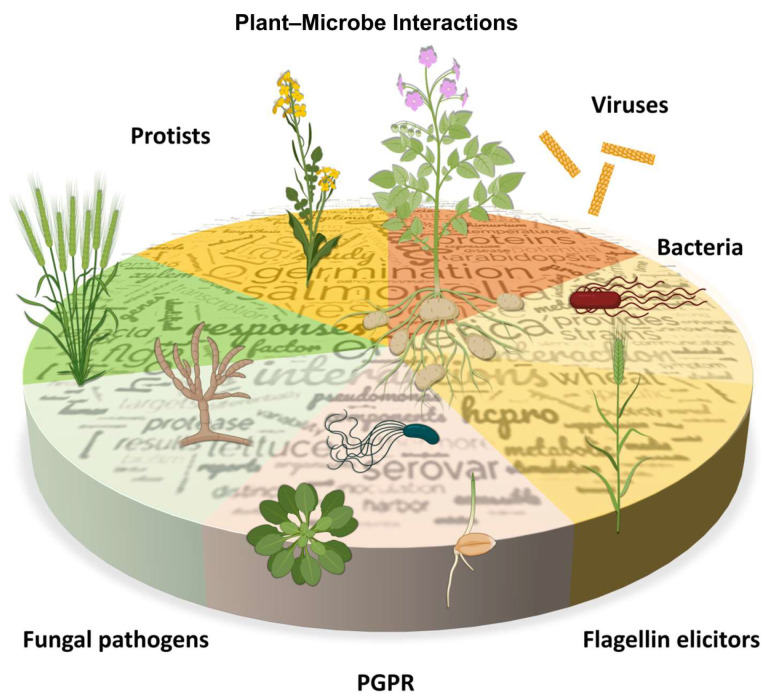
Overview of the diverse plant–microbe interactions explored in this Special Issue. The word cloud within the chart provides a visual representation of the most frequently occurring keywords found in the abstracts.
